# Physical Exercise Potentially Targets Epicardial Adipose Tissue to Reduce Cardiovascular Disease Risk in Patients with Metabolic Diseases: Oxidative Stress and Inflammation Emerge as Major Therapeutic Targets

**DOI:** 10.3390/antiox10111758

**Published:** 2021-11-04

**Authors:** Thembeka A. Nyawo, Carmen Pheiffer, Sithandiwe E. Mazibuko-Mbeje, Sinenhlanhla X. H. Mthembu, Tawanda M. Nyambuya, Bongani B. Nkambule, Hanél Sadie-Van Gijsen, Hans Strijdom, Luca Tiano, Phiwayinkosi V. Dludla

**Affiliations:** 1 Biomedical Research and Innovation Platform, South African Medical Research Council, Cape Town 7505, South Africa; thembeka.nyawo@mrc.ac.za (T.A.N.); carmen.pheiffer@mrc.ac.za (C.P.); sinenhlanhla.mthembu@mrc.ac.za (S.X.H.M.); 2 Centre for Cardiometabolic Research in Africa (CARMA), Division of Medical Physiology, Faculty of Medicine and Health Sciences, Stellenbosch University, Cape Town 7505, South Africa; hsadie@sun.ac.za (H.S.-V.G.); jgstr@sun.ac.za (H.S.); 3 Department of Obstetrics and Gynaecology, Faculty of Health Sciences, University of Pretoria, Pretoria 0001, South Africa; 4 Department of Biochemistry, North-West University, Mafikeng Campus, Mmabatho 2735, South Africa; 36588296@nwu.ac.za; 5 Department of Health Sciences, Faculty of Health and Applied Sciences, Namibia University of Science and Technology, Windhoek 9000, Namibia; mnyambuya@nust.na; 6 School of Laboratory Medicine and Medical Sciences, College of Health Sciences, University of KwaZulu-Natal, Durban 4000, South Africa; nkambuleb@ukzn.ac.za; 7 Department of Life and Environmental Sciences, Polytechnic University of Marche, 60131 Ancona, Italy; l.tiano@univpm.it

**Keywords:** epicardial adipose tissue, oxidative stress, inflammation, cardiovascular disease, exercise, physical activity

## Abstract

Excess epicardial adiposity, within a state of obesity and metabolic syndrome, is emerging as an important risk factor for the development of cardiovascular diseases (CVDs). Accordingly, increased epicardial fat thickness (EFT) implicates the exacerbation of pathological mechanisms involving oxidative stress and inflammation within the heart, which may accelerate the development of CVDs. This explains increased interest in targeting EFT reduction to attenuate the detrimental effects of oxidative stress and inflammation within the setting of metabolic syndrome. Here, we critically discuss clinical and preclinical evidence on the impact of physical exercise on EFT in correlation with reduced CVD risk within a setting of metabolic disease. This review also brings a unique perspective on the implications of oxidative stress and inflammation as major pathological consequences that link increased EFT to accelerated CVD risk in conditions of metabolic disease.

## 1. Introduction

Epicardial adipose tissue (EAT), due to its close proximity to the heart, is known to affect the cardiovascular system by releasing excess lipids such as low-density lipoproteins (LDL), as well as adipokines such as interleukin (IL)-6 that can elicit an undesired pro-inflammatory response, subsequently prompting atherosclerosis and vascular endothelial dysfunction [1,2,3]. In clinical research, measuring EAT volume or mass using non-invasive imaging procedures such as computed tomography or magnetic resonance imaging and echocardiography has proved essential to assess potential cardiovascular disease (CVD) risk [4]. Increased epicardial fat thickness (EFT), which may occur concurrently with elevated parameters of oxidative stress and inflammation, has been shown to be a useful marker for increased CVD risk in patients with metabolic syndrome [5,6,7,8,9]. This has increased research efforts to establish whether reducing EFT expansion may be a feasible strategy to lower CVD risk in individuals with metabolic abnormalities. Several reviews on the feasibility of using physical exercise to reduce EFT and protect against CVDs have been published. For example, in 2015, Rabkin and Campbell [10] conducted a systematic review and meta-analysis comparing interventions such as exercise, diet or bariatric surgery for their role in reducing EFT to lower CVD risk. They revealed that diet and bariatric surgery could markedly reduce EAT, but this was not achieved with exercise. Moreover, reduction in body mass index (BMI) was significantly associated with reduced EAT for diet-based interventions. In other review papers, inflammation plays a central role in the pathophysiological mechanisms linked with EAT [3,11].

More review articles published in 2020 [12] and early 2021 [13,14] have supported the notion that exercise, together with a restricted diet, bariatric surgery and some pharmaceutical interventions, can reduce EAT volume to improve cardiac function. Indeed, these reviews have further highlighted the impact of physical exercise on reducing EFT to attenuate pathological mechanisms, such as those involving inflammation, to improve cardiac function within a setting of metabolic disease. Here, we undertook a comprehensive approach to update and discuss evidence from both preclinical and clinical studies on the impact of physical exercise on EAT and associated CVD risk. Beyond giving a general overview on increased EAT mass and volume and its impact on CVDs, the current review brings a unique perspective on oxidative stress and inflammation as major pathological consequences that link increased EAT mass and volume to accelerated CVD risk in conditions of metabolic disease.

## 2. General Overview of EFT and Its Impact on CVDs

EAT is considered a visceral fat depot that surrounds 80% of the heart’s surface [15]. The multifaceted functional characteristics of EAT include mechanical, metabolic, thermogenic, and endocrine properties to support heart function [15]. In the physiological state, EAT plays a pivotal role in cardiac function, primarily due to its ability to take up and metabolize excess lipids, thus preventing atherosclerotic plaque formation [16]. However, during pathological conditions resulting from oxidative stress or inflammation, EAT may cause detrimental effects to the heart. Similar to other fat depots, EAT is affected by exercise [13,14]. Notably, beneficial effects of exercise on the heart include reducing the EAT mass and volume, which may be followed by increased mitochondrial biogenesis and anti-inflammatory effects that ultimately influence cardiac function [17]. Different exercise stress tests are often used to determine or monitor cardiac functional capacity, and these have been used as an independent indicator of cardiac events [18,19]. For example, treadmill exercise tests are regularly applied in sports and occupational medicine for disease stratification and to monitor response to treatment [19]. Negative exercise stress tests indicate absence of a condition, while positive tests point to diagnosis of coronary artery disease (CAD) [19,20]. Interestingly, it has been observed that patients with coronary slow flow phenomenon display negative exercise stress electrocardiography that correlates with enhanced left ventricular function, while those with a positive exercise stress test exhibit impaired left ventricular function [21]. Accordingly, several studies have reported on the link between EFT and CVD risk in response to the treadmill stress test (Table 1).

Briefly, in patients with established coronary microvascular dysfunction, Sengul et al. [22] found that increased EFT was associated with altered blood pressure responses to exercise stress testing, a risk factor for hypertension. Parsaei et al. [7] reported that EFT was significantly higher in patients with positive exercise test results, and this was associated with reduced levels of high-density lipoprotein (HDL) cholesterol. Moreover, Katlandur et al. [6] showed that EFT was significantly correlated with the severity and prevalence of CAD in positive exercise stress test patients. Türker Duyuler et al. [23] confirmed the relationship between increased EFT and raised blood pressure during stress testing, although these effects did not affect homocysteine levels. Plasma homocysteine levels are inversely correlated with HDL cholesterol [24] and have been shown to be an independent predictor of CVD by contributing to arterial damage and the formation of blood clots [25]. Gorter et al. [26] found that EFT positively correlated with increased BMI and right ventricular end-diastolic pressure and pulmonary vascular resistance, but inversely associated with maximum oxygen consumption rate (VO_2_-max) and exercise capacity in patients with preserved ejection fraction heart failure. Similarly, Haykowsky et al. [27] reported that patients with preserved ejection fraction heart failure had substantially lower EFT than healthy controls, which correlated with reduced peak oxygen uptake and decreased cardiac function. These findings suggest that increased EFT may not consistently indicate increased CVD risk, especially in older patients with metabolic disease and preserved ejection fraction heart failure. Summarized findings in Table 1 highlight the link between EFT and increased CVD risk in patients with metabolic syndrome or those already presenting with established cardiovascular complications.

**Table 1:** Evidence of the link between epicardial fat thickness and cardiovascular disease risk in response to the treadmill stress test.

Study	Country	Participants	Main Findings
Sengul et al., 2011 [22]	Turkey	32 patients with hypertension, with an average age of 49 years	Epicardial fat thickness (EFT) was associated with altered blood pressure responses to exercise stress testing, a risk factor for hypertension.
Parsaei et al., 2014 [7]	Iran	62 patients with coronary microvascular dysfunction, with an average age of 52 years	EFT was significantly higher in patients with positive exercise test results. Moreover, high-density lipoprotein (HDL) cholesterol levels were significantly lower in patients with positive exercise test.
Fidan-Yaylali et al., 2016 [28]	Turkey	114 obese subjects, with an average age of 41 years	EFT was not associated with autonomic nervous system dysfunction.
Katlandur et al., 2016 [6]	Turkey	45 patients with severe coronary artery disease, with an average age of 62 years	EFT was significantly correlated with the severity and prevalence of coronary artery disease in positive exercise test patients.
Türker Duyuler et al., 2017 [23]	Turkey	40 patients with hypertension, with an average age of 47 years	EFT positively correlated with high blood pressure in response to exercise stress testing, although homocysteine levels were not affected.
Haykowsky et al., 2018 [27]	United States	100 obese patients with heart failure with preserved ejection fraction, with an average age of 66 years	Lower EFT was consistent with peak oxygen uptake during exercise stress testing and was negatively associated with decreased cardiac function.
Cho et al., 2017 [29]	South Korea	64 patients with metabolic syndrome, with an average age of 52 years	Increased EFT was correlated with reduced heart rate recovery during exercise stress testing and severe liver steatosis.
Sugita et al., 2020 [30]	Japan	176 patients with type 2 diabetes, with an average age of 65	Increased EFT was positively associated with left ventricular structural and functional abnormalities and exercise intolerance.
Gorter et al., 2020 [26]	Netherlands	75 patients with heart failure with preserved ejection fraction, with an average age of 74 years	EFT correlated with high body mass index, as well as increased right ventricular end-diastolic pressure and pulmonary vascular resistance, but inversely correlated with maximum oxygen consumption rate (VO_2_-max) and exercise capacity.

## 3. Oxidative Stress and Inflammation as Major Pathological Factors Linking EFT to Increased CVD Risk

**Figure 1:**
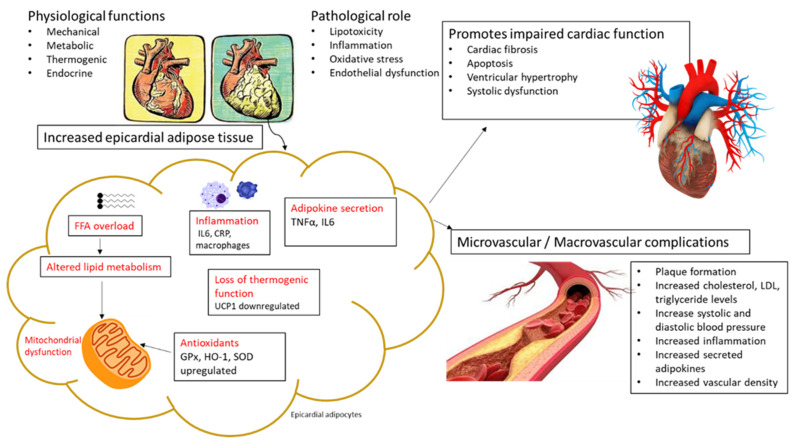
An overview of pathological mechanisms linking increased epicardial adipose tissue (EAT) with the detrimental effects of oxidative stress and inflammation. Briefly, the expansion of EAT is consistent with raised heart weight, and this may be associated with free fatty acid (FFA) overload and impaired lipid metabolism and mitochondrial dysfunction, as evident in some preclinical and clinical studies summarized in the current review. As such, prominent markers of oxidative stress and inflammation that are consistently altered within EAT and that are also detected in the myocardial tissue and in circulation, include glutathione peroxidase (GPx), heme oxygenase 1 (HO-1), superoxide dismutase (SOD), C-reactive protein (CRP), interleukin (IL)-6, and tumor necrosis factor alpha (TNF-α). Notably, the expression levels of some antioxidant genes such as GPx, HO-1, and SOD were increased within EAT in some patients at risk of cardiovascular disease (CVD), indicating a possible adaptive response against oxidative damage. Overall, beyond the detrimental effects of oxidative stress and inflammation, other factors that may be induced through high fat diet feeding, such as dysfunctional adipose tissue and altered thermogenesis, can contribute to the development of cardiovascular complications and increased CVD risk in conditions of metabolic disease. Uncoupling protein 1 (UCP1), Low density lipoprotein (LDL).

Understanding how EAT expansion during obesity or in patients with metabolic disease is related to increased CVD risk (as highlighted in Table 1) has garnered considerable interest in recent years. Key to this is the elucidation of the intricate pathological or molecular mechanisms that are implicated in cardiac abnormalities within EAT. Oxidative stress and inflammation are well-established pathological features that are associated with the development and progression of various metabolic complications, which may or may not be linked with EAT expansion [31,32,33]. The accumulation of triglycerides and lipid metabolites such as ceramides within EAT contributes to lipotoxicity and mitochondrial dysfunction, subsequently causing elevation in markers of oxidative stress and an undesired inflammation that drive cardiac hypertrophy, as represented in Figure 1. In fact, a number of studies have linked increased circulating levels of oxidative products such as thiobarbituric acid reactive substances (TBARS) and pro-inflammatory markers such as C-reactive protein (CRP) with CVD risk [34,35,36]. Likewise, several reviews have explored the pathological link between oxidative stress, inflammation and EFT in individuals at risk of CVD. In 2011, Sacks and Fain [37] proposed that increased EAT volume may be an indicator of acute myocardial infarction, and that increased inflammatory cells and pro-inflammatory actions within EAT are associated with oxidative stress in people with impaired metabolic function. In an expert review published in 2017, Wong et al. [38] discussed data from basic science and translational studies and concluded that an undesired pro-inflammatory response and raised markers of oxidative stress are involved in the pathology linking EAT with CVD risk. Others [39,40,41,42] have emphasized that lipid metabolism in cardiomyocytes appears to be primarily influenced by ectopic fat deposits, leading to endoplasmic reticulum stress, mitochondrial dysfunction and oxidative stress, with an exacerbated inflammatory response and cell death. A few authors [33,43,44] have suggested that increased cardiac oxidative stress and inflammation within an obese state may lead to heart failure, while therapies that are able to attenuate these conditions may diminish the risk of developing cardiovascular complications. This information highlights the importance of understanding the implications of oxidative stress and inflammation in driving the pathological impact of EAT in patients with metabolic syndrome.

Table 2 gives an overview of preclinical studies reporting on the link between oxidative stress, inflammation, EAT and increased CVD risk. Briefly, Company et al. [45] demonstrated that increased EAT volume was correlated with raised heart weight and enhanced messenger ribonucleic acid (mRNA) expression levels of oxidative stress markers, including down-regulation of glutathione peroxidase (GPx), heme oxygenase (HO-1), and superoxide dismutase (SOD) and up-regulation of endothelial nitric oxide synthase (eNOS) in peri-myocardial EAT of pigs with coronary atherosclerosis. Kang et al. [46] reported that EAT was significantly higher in rats fed a high fat diet (HFD), when compared to standard controls. Interestingly, this consequence was correlated with increased myocardial mRNA expression levels of mitochondrial oxidative phosphorylation (OXPHOS) subunit of NDUFB5 (NADH: Ubiquinone Oxidoreductase Subunit B5) and peroxisome proliferator-activated receptor gamma coactivator 1-α (PGC-1 α), and the mitochondrial DNA copy number. Similarly, there were also increased levels of 8-hydroxydeoxyguanosine within the myocardial tissue, which is a known marker consistent with oxidative damage. Besides reporting on the levels of oxidative stress, Xu et al. [47] indicated that increased EAT weight was associated with increased total cholesterol, enhanced expression of matrix metalloproteinase (MMP)2/9 in the aorta and left ventricle, as well as raised pro-inflammatory markers, including serum concentrations of high-sensitivity (hs)-CRP and IL-6 in New Zealand white rabbits fed HFD. Patel et al. [48] showed that loss of angiotensin-converting enzyme (ACE2), a key pathogenic mechanism involved in the development of CVDs, resulted in decreased weight gain but increased glucose intolerance and EAT inflammation, including polarization of EAT resident macrophages into a pro-inflammatory phenotype in mice fed HFD. Thus, from the brief preclinical evidence summarized in Table 2, EAT expansion appearing in conditions of HFD seems to occur consistent with raised markers of oxidative stress and inflammation.

Table 3 further evaluates any correlation between markers of oxidative stress and inflammation, as well as EAT expansion and CVD risk in clinical studies. Apparently, consistent with evidence from preclinical studies (presented in Table 2), clinical data indicate that EAT expansion may occur concomitant with raised markers of oxidative stress and inflammation in patients with metabolic syndrome or those at increased risk of CVD [34,49]. In fact, recently reviewed clinical evidence also infers that measuring EAT may provide an important and reproducible diagnostic tool to stratify patients at risk of heart failure [50]. In addition, elevated pro-inflammatory markers such as CRP and interleukin (IL)-6 are considered reliable biomarkers to indicate increased risk of myocardial injury in patients with T2D at increased risk of developing CVD [35,51,52]. Here, several studies support the notion that impaired lipid metabolism in EAT may be a central feature in the pathological mechanisms that connect EAT with mitochondrial dysfunction, oxidative stress, and an undesired pro-inflammatory response. For example, Sengul et al. [53] and Aydogdu et al. [5] reported that increased EFT correlated with increased waist circumference, elevated LDL-cholesterol, fasting glucose, triglyceride and hs-CRP concentrations and higher systolic and diastolic blood pressure levels in patients with metabolic syndrome. Sacks et al. [54] showed that expanded EAT was accompanied by increased expression of pro-inflammatory and oxidative stress markers such as GPx3 and HO-1 in EAT of patients with CAD. Notably, the increase in antioxidant defense genes with EAT expansion could indicate an essential adaptive response mechanism that is necessary to protect against oxidative stress, as reported elsewhere [55,56,57]. Nonetheless, it is evident that well-known markers of oxidative stress and inflammation such as TBARS, hs-CRP and IL-6 may play a significant role in linking EAT with enhanced CVD risk in conditions of metabolic disease, as reviewed elsewhere [38,58]. Moreover, Chechi et al. [59] observed that EAT biopsies from patients undergoing various heart surgeries display increased expression of genes involved in inflammation, such as tumor necrosis factor (TNF)-α, including the presence of adipose tissue expansion and thermogenic genes such as fatty acid binding protein-4 (FABP4) and uncoupling protein 1. Recently, Zhao et al. [60] showed that the expression of proteins such as serine proteinase inhibitor A3, which are involved in heart failure-related processes including inflammation, oxidative stress, and lipid metabolism, are altered in EAT of patients with heart failure. Ultimately, these findings highlight that EAT in the metabolic disease state is associated with impaired expression of genes involved in various pathological mechanisms such as inflammation, oxidative stress, and thermogenesis. Other studies mentioned in this review show that derangements in the molecular signature of EAT during metabolic disease are related to abnormalities in cardiac mitochondrial functional capacity, as well as myocardial structural and functional competencies that may accelerate CVD risk (Table 2 and Table 3). Overall, the current state of evidence suggests that exacerbated oxidative stress and inflammation within EAT during metabolic disorders are major therapeutic targets to improve cardiac function and reduce CVD risk (Figure 1).

**Table 2:** An overview of preclinical studies reporting on the link between oxidative stress, inflammation, epicardial adipose tissue, and increased cardiovascular disease risk.

Author, Year	Country	Preclinical Model	Main Findings
Company et al., 2010 [45]	United States	13 pigs with coronary atherosclerosis, with ages ranging between 10–11 months	Increased epicardial adipose tissue (EAT) weight was correlated with raised heart weight and enhanced mRNA expression levels of oxidative stress markers, including down-regulated glutathione peroxidase, heme oxygenase, superoxide dismutase, and up-regulated endothelial nitric oxide synthase in peri-myocardial EAT
Kang et al., 2015 [46]	Korea	6 Wistar rats fed a high fat diet (HFD) for 10 weeks	EAT was significantly higher in the HFD group when compared to rats fed a standard diet. This correlated with increased myocardial expression levels of mitochondrial oxidative phosphorylation (OXPHOS) subunit NDUFB5 (NADH:Ubiquinone Oxidoreductase Subunit B5) and peroxisome proliferator-activated receptor gamma coactivator 1-α (PGC1-α), and the mitochondrial DNA copy number. There were also increased levels of 8-hydroxydeoxyguanosine within the myocardial tissue, a known marker for oxidative damage
Xu et al., 2015 [47]	China	6 New Zealand white rabbits fed HFD for 12 weeks	Increased EAT weight was associated with increased total cholesterol, enhanced expression of matrix metalloproteinase (MMP)2/9 in the aorta and left ventricle, as well as raised high-sensitivity CRP and IL-6 serum levels
Patel et al., 2016 [48]	Canada	Angiotensin-converting enzyme 2 (ACE2) knockout mice were fed HFD from weaning to 6 months of age	Loss of ACE2 resulted in decreased weight gain but increased glucose intolerance, EAT inflammation, and polarization of EAT resident macrophages into a pro-inflammatory phenotype in response to HFD

**Table 3:** An overview of clinical studies reporting on the link between oxidative stress, inflammation, epicardial adipose tissue, and increased cardiovascular disease risk.

Author, Year	Country	Characteristic Features of Participants	Main Findings
Salgado-Somoza et al., 2010 [49]	Spain	55 patients with metabolic syndrome undergoing heart surgery, with an average age of 71 ± 9 years	Higher reactive oxygen species production and differential expression of oxidative stress related genes catalase, glutathione S-transferase P, and protein disulfide isomerase in epicardial adipose tissue (EAT) compared to subcutaneous adipose tissue
Wilund et al., 2010 [34]	United States	9 patients on maintenance hemodialysis at increased risk of cardiovascular disease, with an average age of 59 ± 4.9 years	Enhanced EAT correlated with increased serum markers of thiobarbituric acid reactive substances, a marker of oxidative stress. This was also consistent with increased serum lipids and inflammatory markers, C-reactive protein (CRP), and interleukin (IL)-6
Sacks et al., 2011 [54]	United States	16 patients with severe stable coronary artery disease, with age range between 40 and 60 years	A depot specific increase in expression of pro-inflammatory, redox, endothelial cell, and angiogenic genes; additionally, glutathione peroxidase 3, heme oxygenase, and IL-8 gene expression were increased in EAT
Sengul et al., 2011 [53]	Turkey	40 patients with metabolic syndrome, with an average age of 48 years	Increased epicardial fat thickness (EFT) positively correlated with increased waist circumference, serum levels of total and low-density lipoprotein (LDL)-cholesterol, fasting glucose concentrations, triglycerides, systolic and diastolic blood pressure levels, hs-CRP, and insulin resistance
Aydogdu et al., 2015 [5]	Turkey	30 patients with subclinical hypothyroidism, with an average age of 37 years	EFT and increased oxidative stress index correlated with body fat percentage, serum levels of total cholesterol, LDL, and diastolic blood pressure
Chechi et al., 2017 [59]	Canada	EAT biopsies from patients undergoing various heart surgeries, with an average age of 64 years	EAT biopsies revealed increased expression of genes involved in inflammation such as tumor necrosis factor-α (TNF-α), as well as the presence of adipose tissue expansion and thermogenic genes such as fatty acid binding protein-4 (FABP4) and uncoupling protein 1 (UCP1)
Zhao et al., 2020 [60]	China	5 patients with heart failure	Proteomic analysis of EAT revealed increased levels of proteins involved in inflammation and lipid metabolism, including serine proteinase inhibitor A3 and fatty acid synthase

## 4. Evidence on the Impact of Physical Exercise on EFT and CVD-Related Markers

The rising trends in metabolic disorders, especially the state of overweight and obesity, contribute significantly to impaired metabolic function through adipose tissue expansion [61,62]. A sedentary lifestyle, which is characterized by little or no physical exercise, is known to promote ectopic lipid accumulation and cardiac deterioration, partially through expansion of EAT [61,62]. Accordingly, it is suggested that interventions that can reduce excessive body fat accumulation, including EAT, can lower CVD risk [13,63]. As such, clinical studies have markedly increased reporting on the impact of moderate to intensive physical exercise on EFT in individuals at risk of developing cardiovascular complications. Such studies have predominantly involved adult obese subjects or those with established cardiovascular complications. Some of these studies have been published in developing countries such as India or China that are plagued by rapidly rising cases of noncommunicable diseases such as type 2 diabetes (T2D) [64], whereas most publications are from developed countries such as the United States and in Europe. Notably, there is limited literature, if any, reporting on the impact of physical exercise on EFT in Africa. Nonetheless, data summarized in Table 4 and Table 5 supports the beneficial effects of different forms of physical exercise, ranging from moderate to high intensity, in reducing EFT and improving metabolic parameters in patients at increased CVD risk.

Briefly, findings in obese subjects including those with a cluster of metabolic complications such as glucose intolerance, hypertension or CAD, support the beneficial effects of short-term physical exercise (<3 months) in reducing EFT, subsequently influencing markers that are essential in determining cardiac function (Table 4). For example, Kahl et al. [63] reported that exercise training for 6 weeks reduced EFT, along with improving metabolic factors such as body weight, BMI, and HDL levels in patients with metabolic syndrome, especially those characterized by major depressive disorder. Similarly, Wu et al. [65] demonstrated that aerobic steps that consisted of 3 to 5 sessions per week for 3 months significantly reduced EFT, BMI, waist circumference, and visceral fat in obese subjects. To build on these findings, Honkala et al. [66] reported that high-intensity interval training and moderate-intensity continuous training for 2 weeks effectively reduced EAT mass in subjects with defective glucose tolerance. Interestingly, this study showed that high intensity training appeared superior in improving aerobic capacity and whole-body insulin sensitivity. Other studies on the short-term impact of high intensity physical exercise, either for 3 weeks [67] or 8 weeks [68], demonstrated that reduced EFT was linked with muscular endurance and improved flow-mediated dilation in females with obesity or patients with hypertension, respectively.

Clinical evidence presented in Table 5 evaluated the impact of long-term (≥3 months) physical exercise on EFT expansion in correlation with CVD risk in patients with metabolic diseases (Table 5). Interestingly, although they did not observe a change in epicardial fat volume or cardiac function in diabetic individuals, Jonker et al. [69] reported that moderate to intensive exercise for 6 months could effectively reduce levels of hepatic triglycerides, visceral abdominal fat, and paracardial fat (a thoracic mesenchyme-derived fat pad located superior to the visceral pericardium and around the blood vessels). It is important to note that reduced hepatic triglycerides, visceral abdominal fat, and paracardial fat are associated with decreased CVD risk in diabetic individuals [2]. As such, Serrano-Ferrer et al. [70] demonstrated that 6 months of resistance and endurance exercise could reduce EFT and improve left ventricular strains and inflammatory profiles (increased adiponectin and reduced TNF-α) in adult patients with metabolic syndrome. Notably, also indicating the potential role of long-term physical exercise in targeting EFT to improve inflammatory status, others have reported that 3 months of aerobic exercise training [71] or resistance training [72] remains effective in decreasing EFT volume or EAT mass, concomitant with reducing the levels of hs-CRP as well as known CVD risk indices such as resting heart rate, LDL levels, and total cholesterol levels in patients with metabolic syndrome. Generally, these results suggest that enhanced adiposity and increased EFT in individuals with metabolic syndrome or already presenting with CAD are consistent with elevated inflammation and lower cardiac efficiency. In turn, by reducing body weight, vigorous physical exercise can significantly reduce EFT and, as a result, improve metabolic function, attenuate an undesired pro-inflammatory response, and enhance cardiac efficiency.

Additional clinical studies have also reported on the positive effects of the long-term effects of physical exercise on the reduction of EFT in patients with heart failure or diagnosed with CVDs (Table 5). Jo et al. [73] demonstrated that 3 months of exergame and treadmill exercise showed similar effects in reducing EFT and improving markers of cardiorespiratory fitness and endothelial function, including VO_2_-max and flow-mediated dilation in postmenopausal women with high CVD risk. Zhang et al. [74] revealed that 90 min of Tai Chi exercise daily for 3 months could reduce EFT, lower heart rate, and increase the quality of life of patients with heart disease. Mechanistically, this evidence was supported by reduced levels of microRNA (miR)-126, which is one of the prominent markers of inflammation related with elevated CVD risk [75,76]. Markers of inflammation, especially the emerging role of microRNAs in metabolic diseases and CVDs, are attracting interest to develop novel therapeutics [77,78]. Exercise has also been shown to decrease mitogen activated protein kinase (MAPK) activity, one of the signaling pathways involved in cardiac hypertrophy and heart failure, and inflammatory responses in aortas of adult male Sprague-Dawley rats [79]. Taken together, these findings highlight that regardless of the type of exercise intervention, in addition to reducing EFT, long-term (≥3 months) exercise has various beneficial effects on the cardiovascular system, in part by attenuating systematic inflammation and improving cardiac efficiency in conditions of metabolic syndrome. Interestingly, others indicated that physical activity in combination with caloric restriction for 4 months could also reduce EFT; however, there were no significant differences in cardiometabolic profile in patients with T2D [80]. However, such evidence is still limited, and additional studies are necessary to assess the therapeutic impact of combining physical exercise with caloric restriction.

**Table 4:** An overview of studies on the impact of physical exercise for less than 3 months on epicardial adipose tissue thickness in individuals at risk of cardiovascular disease.

Study	Country	Study Size and Population	Main Findings
Kahl et al., 2016 [63]	Germany	20 participants with major depressive disorder, with an average age of 44 years	Six weeks of moderate intensity exercise training reduced epicardial fat thickness (EFT) and improved metabolic factors such as body weight, body mass index, and high-density lipoprotein
Wu et al., 2016 [65]	Taiwan	39 obese subjects, with an average age of 39 years	Aerobic steps that consisted of 3 to 5 sessions per week for 3 months significantly reduced EFT, body mass index, waist circumference, and visceral fat
Honkala et al., 2017 [66]	Finland	16 subjects with defective glucose tolerance, with ages ranging between 40 and 55 years	High-intensity interval training and moderate-intensity continuous training for 2 weeks effectively reduced epicardial adipose tissue mass. However, high intensity training appeared superior in improving aerobic capacity and whole-body insulin sensitivity
Fernandez-del-Valle et al., 2018 [67]	United States	6 young females with obesity, with an average age of 22 years	Epicardial and paracardial fat volumes were reduced by 3 weeks of high intensity, moderate-volume muscular endurance resistance training
Jo et al., 2020 [68]	South Korea	17 patients with hypertension, with an average age of 50 years	High and moderate intensity training for 8 weeks significantly reduced EFT and improved flow-mediated dilation

**Table 5:** An overview of studies on the impact of physical exercise for 3 months or more on epicardial adipose tissue thickness in individuals at risk of cardiovascular disease.

Study	Country	Study Size and Population	Main Findings
Jonker et al., 2013 [69]	Netherlands	12 patients with type 2 diabetes (T2D), with an average age of 46 years	Six months of moderate to intensive exercise decreased paracardial fat volume hepatic triglyceride content and visceral abdominal fat. However, cardiac function was unaffected
Serrano-Ferrer et al., 2016 [70]	France	87 patients with metabolic syndrome, with an average age of 59 years	Six months of resistance and endurance exercise reduced epicardial fat thickness (EFT) and improved left ventricular strains and inflammatory profiles (increased adiponectin and reduced tumor necrosis factor alpha expression)
Bairapareddy et al., 2018 [71]	India	66 overweight subjects, with age ranging between 20 and 45 years	Three months of aerobic exercise training resulted in a significant reduction in EFT and decreased body weight and high sensitivity C-reactive protein levels
Christensen et al., 2019 [72]	United States	50 physically inactive participants with abdominal obesity, with an average age of 39 years	Three months of endurance and resistance training reduced epicardial adipose tissue (EAT) mass, resting heart rate as well as cardiometabolic parameters such as low-density lipoproteins and total cholesterol
Jo et al., 2020 [73]	South Korea	44 postmenopausal women with high cardiovascular disease risk, with an age range between 57–62 years	Three months of exergame and treadmill exercise showed similar effects in reducing EFT and improving markers of cardiorespiratory fitness and endothelial function, including VO_2_-max and flow-mediated dilation
Zhang et al., 2020 [74]	China	36 patients with heart disease, with an average age of 62 years	Ninety minutes of Tai Chi exercise daily for three months decreased EAT volume and heart rate and improved quality of life. This was concomitant with lowered levels of microRNA (miR)-126, mitogen-activated protein kinase, c-Jun *N*-terminal kinases, and extracellular signal-regulated kinase
Leroux-Stewart et al., 2021 [80]	Canada	26 patients with T2D, with an average age of 58 years	Physical activity in combination with caloric restriction for 4 months was effective in reducing EFT and total fat mass; however, there were no significant differences in cardiometabolic profile

## 5. Summary and Conclusions

Non-communicable diseases, especially metabolic syndrome, contribute significantly to the rising global disease burden [81]. Increasing research has been directed to identifying the precise pathophysiological mechanisms that are implicated in the development of diverse metabolic complications, including gestational diabetes, an important feature predominantly seen in pregnant patients with T2D [82,83]. Seemingly, determining this aspect remains crucial for early disease diagnosis, especially to identify potential therapeutics that could be effectively used to protect against or even slow disease progression. As such, excessive adiposity in major organs including the heart, usually seen in conditions of obesity or metabolic syndrome, has been consistently linked to CVD [16,84]. Accumulative research is currently underway to determine how EFT affects cardiac efficiency in individuals at risk of CVDs [5,42,85]. From the current review, we were able to summarize the use of treadmill exercise tests in identifying individuals at risk of CVD. Moreover, it was evident that increased EFT was constantly associated with reduced HDL-cholesterol, raised triglycerides, and left ventricular structural and functional abnormalities that occurred concurrent with the severity and prevalence of cardiovascular complications (Table 1). Inferring that increased EFT is strongly associated with increased CVD risk, it may be a reliable therapeutic target to improve cardiac function in individuals with metabolic syndrome.

Although currently used therapeutic drugs such as metformin and statins can effectively reduce blood glucose or lipid levels to prolong the lives of patients with metabolic syndrome, the long-term use of these drugs has been associated with several limitations including toxicity, ultimately leading to increased CVD risk [86,87,88]. Certainly, from evidence presented in the current review (Table 2 and Table 3), we hypothesize that increased EFT occurs concurrently with raised circulating levels of TBARS, hs-CRP, and IL-6, indicating oxidative stress and chronic low-grade inflammation. This consequence was associated with impaired lipid metabolism, mitochondrial dysfunction within the myocardium, reduced myocardial respiration, increased cardiac steatosis, and worsened cardiac function in conditions of metabolic syndrome [48,59]. These findings suggest that increased EFT is significantly associated with increased CVD risk; thus, it remains essential to develop therapies that target reduction of EFT beyond the whole-body fat, in an effort to lower CVD risk.

**Figure 2:**
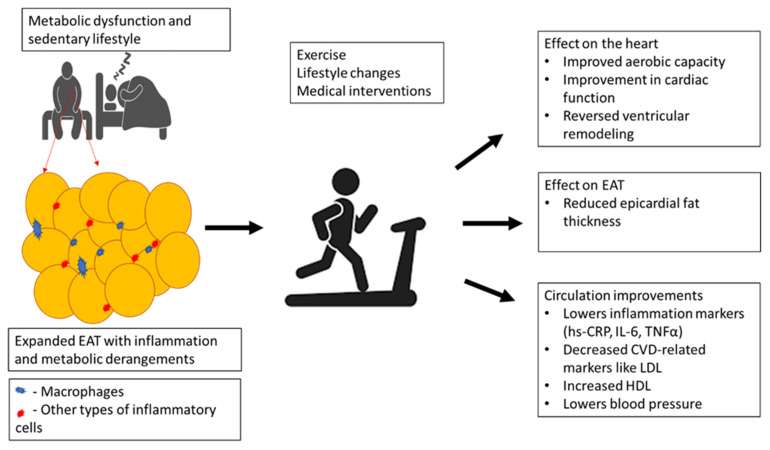
An overview of how physical exercise may contribute to lowered cardiovascular disease (CVD) risk by improving metabolic function and myocardial maximal oxygen uptake, especially by reducing epicardial adipose tissue (EAT) thickness in correlation with markers of inflammation in patients with obesity or metabolic syndrome. Briefly, physical exercise can reduce EAT volume to improve myocardial aerobic capacity, lower blood pressure, and reverse the myocardial remodeling process, in part by blocking low-density lipoprotein (LDL) and enhancing high-density lipoprotein (HDL) levels. This is also consistent with decreasing markers of inflammation such as high sensitivity-C-reactive protein (hs-CRP), interleukin (IL)-6, and tumor necrosis factor alpha (TNF-α).

Similar to weight gain, it is currently understood that EFT can be exacerbated by several modifiable risk factors such as a sedentary lifestyle, stress, and an unbalanced diet [42,85]. Studies have demonstrated that physical activity or exercise intervention provides an effective non-invasive strategy for reducing EFT that may also exert beneficial effects on the cardiovascular system [14,71]. In agreement, data from clinical studies summarized in the current review (Table 4 and Table 5) support the notion that vigorous or endurance exercise can improve metabolic function by decreasing weight gain and BMI, and in the process also lower EFT in patients with metabolic syndrome. Moreover, it was observed that reduction in EFT was accompanied by a decrease in inflammatory markers TNF-α and CRP, a consequence that was linked with improved cardiac aerobic capacity and flow-mediated dilation (Table 5). Figure 2 gives an overview of how physical exercise may contribute to lowered CVD risk by improving metabolic function and myocardial maximal oxygen uptake, especially by reducing EFT in correlation with markers of inflammation in patients with obesity or metabolic syndrome.

In conclusion, evidence from the current review supports the regular use of physical activity or exercise to alleviate complications linked with the progression of metabolic diseases, including reducing EFT, to attenuate the detrimental effects of oxidative stress and inflammation in those at risk of developing cardiovascular complications. Notably, while such beneficial effects of physical exercise are acknowledged, few individuals adhere to these interventions. Thus, research into novel therapies that could target EFT to improve cardiac function is still required.

## Abbreviations

Cardiovascular disease (CVD); epicardial fat thickness (EFT); epicardial adipose tissue (EAT); coronary artery disease (CAD); high-density lipoproteins (HDL); type 2 diabetes (T2D); tumor necrosis factor-alpha (TNF α); flow-mediated dilation (FMD); angiotensin-converting enzyme 2 (ACE2); C-reactive protein (CRP); interleukin 6 (IL6); low-density lipoprotein (LDL); thiobarbituric acid reactive substances (TBARS).
